# Distinct gene networks modulate floral induction of autonomous maize and photoperiod-dependent teosinte

**DOI:** 10.1093/jxb/ery110

**Published:** 2018-04-24

**Authors:** Mark A A Minow, Luis M Ávila, Katie Turner, Elena Ponzoni, Iride Mascheretti, Forest M Dussault, Lewis Lukens, Vincenzo Rossi, Joseph Colasanti

**Affiliations:** 1 Department of Molecular and Cellular Biology, University of Guelph, Guelph, Ontario, Canada; 2 Plant Agriculture Department, University of Guelph, Guelph, Ontario, Canada; 3 Council for Agricultural Research and Economics, Research Centre for Cereal and Industrial Crops, Bergamo, Italy; 4 Research and Development, Canadian Food Inspection Agency, Ottawa, Ontario, Canada

**Keywords:** Autonomous flowering, carbon sensing, circadian clock, floral induction, florigen, gene networks, maize migration, photoperiod

## Abstract

Temperate maize was domesticated from its tropical ancestor, teosinte. Whereas temperate maize is an autonomous day-neutral plant, teosinte is an obligate short-day plant that requires uninterrupted long nights to induce flowering. Leaf-derived florigenic signals trigger reproductive growth in both teosinte and temperate maize. To study the genetic mechanisms underlying floral inductive pathways in maize and teosinte, mRNA and small RNA genome-wide expression analyses were conducted on leaf tissue from plants that were induced or not induced to flower. Transcriptome profiles reveal common differentially expressed genes during floral induction, but a comparison of candidate flowering time genes indicates that photoperiod and autonomous pathways act independently. Expression differences in teosinte are consistent with the current paradigm for photoperiod-induced flowering, where changes in circadian clock output trigger florigen production. Conversely, differentially expressed genes in temperate maize link carbon partitioning and flowering, but also show altered expression of circadian clock genes that are distinct from those altered upon photoperiodic induction in teosinte. Altered *miRNA399* levels in both teosinte and maize suggest a novel common connection between flowering and phosphorus perception. These findings provide insights into the molecular mechanisms underlying a strengthened autonomous pathway that enabled maize growth throughout temperate regions.

## Teaser

Comparison of day-neutral temperate maize and photoperiod-dependent teosinte revealed divergent leaf gene expression networks that implicate different genes of the same gene families in response to flowering.

## Introduction

Temperate maize (*Zea mays* ssp. *mays*) is derived from its wild progenitor, teosinte (*Zea mays* ssp. *parviglumis*) (Doebley, 1990). Maize cultivation spread along a north–south axis from the Balsas river basin in Mexico, extending throughout the Americas. In addition to dramatic morphological modifications during domestication, growth in temperate climates required modern maize to respond to alternative stimuli to trigger flowering (Dorweiler *et al.*, 1993; Studer *et al.*, 2011; Hufford *et al.*, 2012; Hung *et al.*, 2012). The transition from vegetative to reproductive growth is a critical event in the plant life cycle that directly influences yield and fitness. Floral induction can be triggered by environmental signals, such as photoperiod, as well as internal autonomous signals. The integration of these inputs causes the floral transition at the shoot apical meristem (SAM). Unlike teosinte, which depends on short-day (SD) photoperiods, endogenous signals control flowering in temperate maize (Coles *et al.*, 2010). Thus, flowering occurs at the same time regardless of day length, allowing maize to produce grain before a killing frost. The genetic mechanisms underlying autonomous signals in temperate maize remain poorly understood.

Study of model plants, notably *Arabidopsis thaliana*, has delineated floral induction mechanisms that are conserved across species (Koornneef *et al.*, 1991). In Arabidopsis, long-day (LD) photoperiods promote early flowering via circadian clock-controlled production of florigen in leaves. A key component of the LD pathway is *CONSTANS* (*CO*), which encodes a transcription factor that cycles in a circadian rhythm within leaves (Putterill *et al.*, 1995). Under LD photoperiods, CO protein accumulates and directly activates *FLOWERING LOCUS T* (*FT*) expression in the phloem (Suárez-López *et al.*, 2001; Ayre and Turgeon, 2004; Tiwari *et al.*, 2010). FT is a phosphatidylethanolamine-binding protein (PEBP) that acts as a mobile florigen (Araki *et al.*, 1998; Corbesier *et al.*, 2007). FT translocates through the phloem to the SAM where it interacts with FLOWERING LOCUS D (FD), a bZIP transcription factor (Abe *et al.*, 2005; Wigge *et al.*, 2005). The FT–FD complex directly activates expression of *APETALA1* (*AP1*) and other targets to establish reproductive meristem identity (Mandel *et al.*, 1992; Weigel *et al.*, 1992). Loss of *CO* or *FT* function causes late flowering under LD conditions, but has little effect on flowering under non-inductive SD conditions (Koornneef *et al.*, 1991).

*FT* homologues with florigenic function are also found in monocotyledonous SD plants (Kojima *et al.*, 2002; Danilevskaya *et al.*, 2010; Krieger *et al.*, 2010). The maize *PEBP* gene family, known as *Zea mays CENTRORADIALIS* (*ZCN*), includes the experimentally validated florigen, *ZCN8* (Lazakis *et al.*, 2011; Meng *et al.*, 2011). Maize possesses an orthologous bZIP transcription factor gene, *DELAYED FLOWERING1* (*DLF1*), that likewise influences flowering time (Muszynski *et al.*, 2006). In teosinte, *ZCN8* is expressed at high levels upon SD induction, and some temperate inbreds retain a residual photoperiodic response (Lazakis *et al.*, 2011; Meng *et al.*, 2011). Loss of an obligate photoperiod requirement in temperate maize is partially attributed to a loss of LD-triggered circadian clock repression of flowering. Evidence suggests that loss of the negative floral regulator, *Zea mays CO*, *CO-LIKE TIMING OF CAB1 PROTEIN DOMAIN* (*ZmCCT*), contributed to the northward expansion of maize (Hung *et al.*, 2012; Yang *et al.*, 2013). Other clock-associated genes, including *CONSTANS1* (*CONZ1*) and *GIGANTEA1* (*GI1*), have minor effects on flowering in temperate maize (Miller *et al.*, 2008; Bendix *et al.*, 2013).

Small RNAs (sRNAs) are regulators of diverse components of plant growth and development, including flowering time. Conserved miRNAs regulate genes that influence autonomous control of flowering (Aukerman and Sakai, 2003; Wu and Poethig, 2006). *miR156* and *miR172* are intertwined antagonists of a conserved age-sensing network (Wu *et al.*, 2009; Cho *et al.*, 2012). During development, *miR156* levels decrease and allow for elevated expression of *miR172*, which down-regulates *APETELA2* (*AP2*) floral repressors (Aukerman and Sakai, 2003). A similar gene regulatory network has been described in maize. The dominant mutant *Corngrass1* (*Cg1*) allele overexpresses a *miR156* gene, and a *miR172* homologue down-regulates *AP2*-like genes including *GLOSSY15* and *RELATED TO AP2.7* (*RAP2.7*) (Lauter *et al.*, 2005; Chuck *et al.*, 2007; Salvi *et al.*, 2007). Interplay between miRNA-mediated gene regulation and other modes of floral induction remains unstudied in maize.

The predominant autonomous floral inductive pathway of temperate maize shares little similarity with characterized autonomous pathways in dicots (Colasanti and Coneva, 2009). The *indeterminate1* gene (*id1*) encodes a transcriptional regulator that controls autonomous floral induction in maize (Colasanti *et al.*, 1998). Loss of *id1* function disrupts the autonomous pathway and severely delays the floral transition (Colasanti *et al.*, 1998). The *id1* gene is expressed exclusively in developing immature leaves (ILs), but loss of *id1* function has no overt effect on leaf morphology. However, *id1* mutant mature leaves (MLs) have increased levels of sucrose and starch, and modified energy metabolism (Coneva *et al.*, 2012). Although the mechanism is unknown, ID1 is thought to be involved in sensing whole-organism carbon status and conveying readiness to flower (Coneva *et al.*, 2007, 2012; Wong and Colasanti, 2007). Carbohydrate sensing is associated with autonomous control of flowering in other plants (Corbesier *et al.*, 1998; Moore *et al.*, 2003; Wahl *et al.*, 2013).

The close genetic relationship between teosinte and temperate maize provides a unique opportunity to study how autonomous floral control evolved from an obligate photoperiodic ancestor. Photoperiod and autonomous pathways, despite responding to different stimuli, both rely on leaf-based signals. During maize domestication, an autonomous signal could have mimicked SDs, but could trigger the same downstream photoperiodic leaf inductive pathway. Alternatively, an independent and weak autonomous pathway could have been strengthened during domestication to allow for flowering in temperate climates. To investigate how the autonomous flowering pathway dominated the ancestral photoperiod pathway, leaf mRNA and sRNA profiles were compared pairwise between flowering and vegetative teosinte and between flowering and vegetative temperate maize. These comparisons show leaf expression overlap between photoperiodic and autonomously flowering *Z. mays*, revealing leaf events that were conserved during maize domestication. This overlap, however, does not include homologues of established floral regulators. Rather, distinct expression profiles of known floral regulators in maize and teosinte were observed. This suggests that the maize autonomous and photoperiodic gene networks act in parallel and only converge after signal perception at the SAM. These findings provide novel insights into mechanisms that favoured autonomous flowering that supplanted photoperiodic induction, fostering the widespread dissemination of maize.

## Materials and methods

### Plant materials and growth conditions

**Fig. 1.**
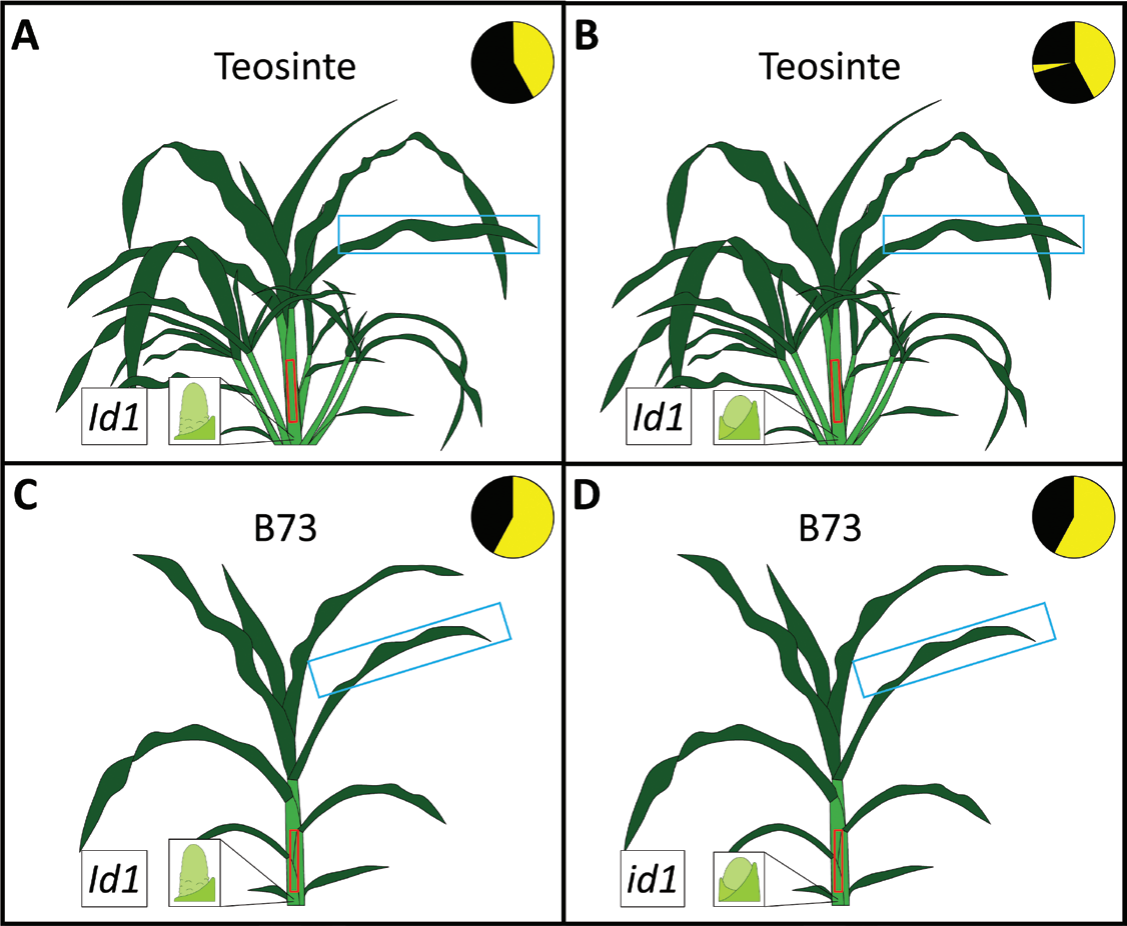
Tissue sampling scheme of reproductive and vegetative teosinte and B73. Teosinte was grown under inductive short-day (SD) conditions (A) and uninductive night-break (NB) conditions (B). B73 inbreds were grown under long-day conditions with either homozygous for (WT) *Id1* (C) or homozygous for mutant *id1* (D) to induce and repress the floral transition, respectively. RNA profiling was conducted on mature leaf (blue box) and the immature leaf whorl above the meristem (red box). Reproductive and vegetative expression changes were first compared (A versus B; C versus D) within subspecies, followed by a comparison of expression changes between teosinte and B73.

Maize and teosinte plants were grown as described previously (Mascheretti *et al.*, 2015) (Fig. 1). *Zea mays* spp. *parviglumis* plants were grown under non-inductive LD conditions (14 h/10 h; light/dark) and then induced to flower by exposure to SD conditions (10 h light/14 h dark) or flowering was inhibited by a 1 h night-break (NB) treatment in the middle of the dark period. The *id1-m1* allele was backcrossed 11 times into *Z. mays* spp. *mays* inbred B73, and segregating flowering *Id1*^*+*^ (WT) and non-flowering plants (*id1-m1*) at the V7 stage were genotyped and used for RNA isolation as previously described (Wong and Colasanti, 2007). Temperate maize plants were grown under LD photoperiods (14 h day/10 h night).

### mRNA profiling and binding site prediction

Total RNA was extracted from three replicates of frozen tissues using the MirVana kit (Ambion) following the manufacturer’s instructions. Samples (10 µg) of total RNA were treated with 3 U of TURBO DNase (Ambion) for 1 h at room temperature in a final volume of 50 µl, followed by purification with RNA Clean and Concentration-5 column (Zymo Research). RNA quality and concentration were estimated by agarose gel electrophoresis and spectrophotometry. Libraries for next-generation Illumina system directional sequencing of total RNA were prepared with TruSeq Stranded Total RNA with a Ribo-Zero Plant kit (Illumina). Sequencing of 24 total RNA-seq (four sample types, each with three biological replicates) was performed at the Istituto di Genomica Applicata (Udine, Italy) on an Illumina Hiseq2500 platform. RNA-seq paired-end 2 × 100 bp sequences, producing ~20 million paired reads per sample, achieved a total of ~60 million 2 × 100 bp paired-end reads for each sample type (NCBI BioProject accession number PRJNA439244). FastQC was used to ensure reads were of appropriate quality (http://www.bioinformatics.babraham.ac.uk/projects/fastqc/). Differential gene expression was analysed using the Tuxedo software suite (Bowtie2 v2.1.0; TopHat v2.0.11; cufflinks v2.2.1) and aligned against the B73 reference genome (V3.22) (Li *et al.*, 2009; Schnable *et al.*, 2009; Trapnell *et al.*, 2012; Langmead and Salzberg, 2012). One B73 *id1* immature leaf biological replicate produced low read alignment rates (27.85%) and was excluded from analysis. To quantify expression from the *ZCN7* and *ZCN8* loci, strand-specific reads were remapped using Bowtie2 independently for both the *ZCN7* and *ZCN8* coding sequence and for the unspliced sense and antisense transcripts. A reference gene (*GRMZM2G161285*) with stable FPKM (fragments per kilobase of transcript per million mapped reads) values with very low variance (6.94 ± 0.24) was selected and used to standardize reads across all libraries. Reads were called to either *ZCN7* or *ZCN8* based on MapQ values. Reads that mapped completely to exons were excluded in the *ZCN7* and *ZCN8* unspliced sense reads. All Gene Ontology (GO) enrichment was conducted using AgriGO (V5a) (Du *et al.*, 2010). Venn diagrams were generated through Bioinformatics & Evolutionary Genomics (http://bioinformatics.psb.ugent.be/webtools/Venn/). Principal component analysis (PCA) was conducted through SPSS using transcripts within the 10th highest percentile of variance across all samples. Sequences upstream (≤2 kb) of genes that were differentially expressed in *Id1*^*+*^ versus *id1-m1* leaves were employed for *in silico* motif discovery using the Find Individual Motif Occurrences (FIMO) tool and degenerate motif TTTGTCSYWWT (IUPAC nucleotide code) (Grant *et al.*, 2011).

### sRNA profiling

For sRNA-seq, total RNA was prepared and purified as described above and used as template for sRNA libraries prepared with the TruSeq small RNA kit (Illumina). Sequencing of the 24 sRNA-seq libraries (four sample types, each with three biological replicates) was performed on an Illumina Hiseq2500 platform. sRNA-seq single-end 1 × 50 bp read sequencing, producing ~10 million reads per sample, was done, thus obtaining a total of ~30 million 1 × 50 bp single-end reads for each sample type. Shortstack (V3.4) aligned sRNA reads to the B73 reference genome (V3.22) and created sRNA counts within sRNA clusters based upon physical position (default settings) (Axtell, 2013). These clusters were compared with annotated *Z. mays* miRNA loci (miRBase) to quantify miRNA expression (Kozomara and Griffiths-Jones, 2014). The R package ‘baySeq’ was used with the counts produced by Shortstack to determine differential sRNA cluster expression (Hardcastle and Kelly, 2010).

### qPCR of mRNA and miRNA

Confirmation of expression trends was conducted on mRNA and miRNA using quantitative PCR (qPCR) and stem–loop qPCR, respectively. The qPCR and statistical analysis of data were performed as described previously (Mascheretti *et al.*, 2015). Preparation of cDNA and stem–loop qPCR was performed as previously reported using the universal UPL28 probe (Roche) (Varkonyi-Gasic *et al.*, 2007). Expression was normalized to *miR166* for miRNA quantification and to both *MEP* and *UBPC* for mRNA quantification. Primers employed for qPCR and stem–loop qPCR are reported in Supplementary Tables S9 and S10 at *JXB* online.

## Results

### Comparing leaf transcriptomes of maize and teosinte at the floral transition

To examine similarities between photoperiod and autonomous floral inductive pathways, plants were grown in four groups (Fig. 1). Teosinte was grown under SDs to induce flowering and under SD photoperiods with a 1 h NB to maintain vegetative growth (Mascheretti *et al.*, 2015). Plants introgressed into inbred background B73, and segregating normal *Id1*^*+*^ and mutant *id1-m1* alleles were grown under LDs. Normal *Id1*^*+*^ plants (hereafter ‘WT’) and *id1* homozygous mutants represent reproductive and vegetative treatments, respectively (Colasanti *et al.*, 1998). Floral inductive signals produced in photosynthetic MLs are influenced by developmental patterning outside of the SAM in the ILs (Coneva *et al.*, 2007; Mascheretti *et al.*, 2015). Therefore, at the floral transition (V7) in WT B73 and SD teosinte, developing ILs and developed MLs were harvested as previously described and used for mRNA and sRNA expression profiling (Mascheretti *et al.*, 2015). Pairwise comparisons in leaf expression were conducted between flowering and non-flowering groups. Teosinte SD was compared with teosinte NB, and B73 WT was compared with B73 *id1*. Finally, expression differences found in florally induced and uninduced teosinte (SD versus NB) were contrasted with those observed in florally induced and uninduced B73 (WT versus *id1*) to evaluate the overlap between the photoperiod and autonomous pathways in the leaf.

### Teosinte leaf mRNA expression changes at the floral transition highlight alterations in circadian clock and florigen gene expression

**Fig. 2.**
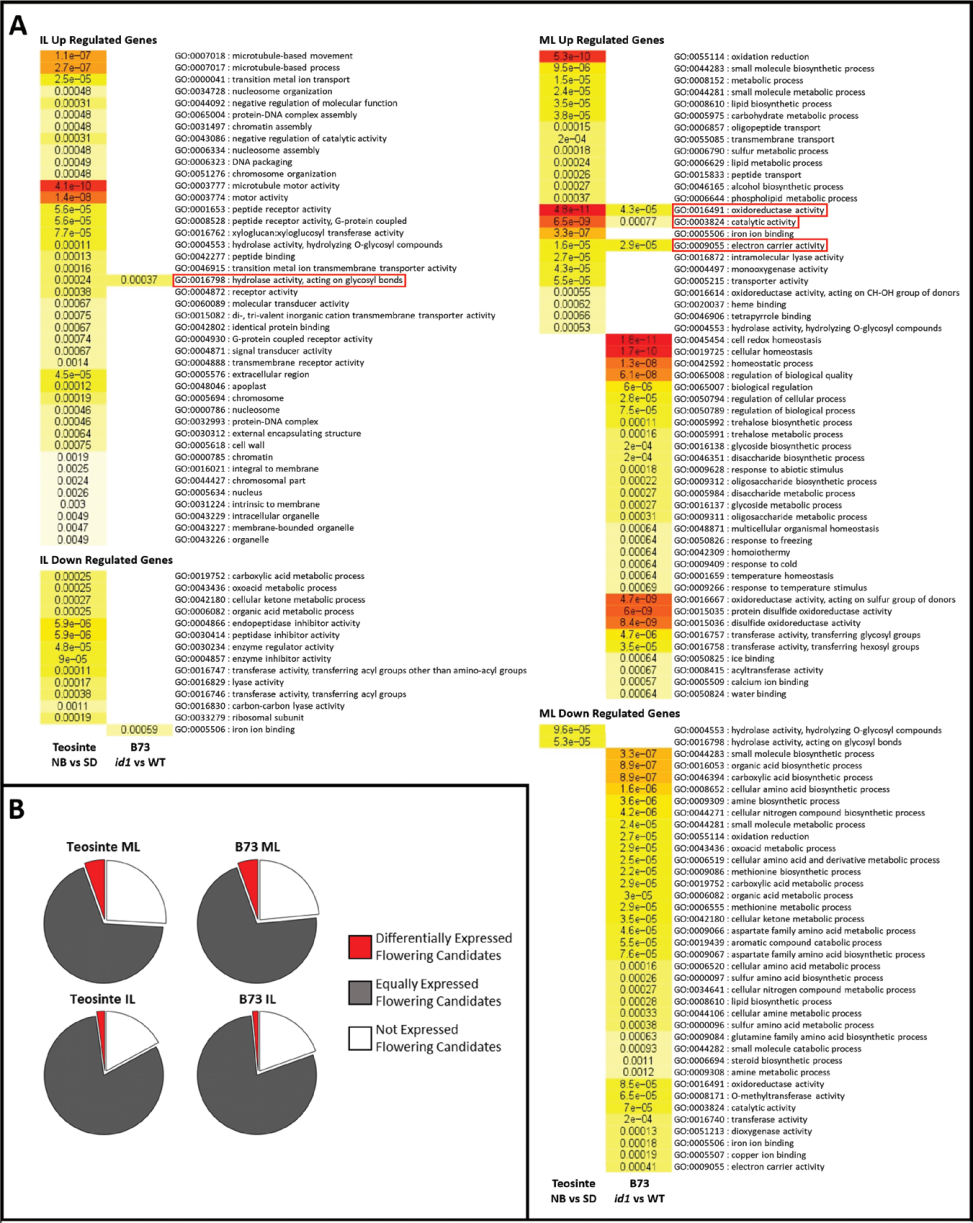
The floral transition in teosinte and B73 results in transcriptional changes enriched in many Gene Ontology (GO) terms and contains candidate floral regulators. (A) Enriched GO terms up- and down-regulated in immature leaves (ILs; left) and mature leaves (MLs; right) in vegetative/reproductive teosinte (night-break; NB versus short-days; SD) and B73 (*id1* versus WT). (B) Proportions of candidate floral regulators that were expressed (grey), differentially expressed (red), or not expressed (white) in vegetative/reproductive teosinte and B73. Shared GO terms are outlined in red, and *P*-values for each ontology term are displayed with a colour reflecting its level of significance.

RNA sequencing from teosinte tissues produced an average of 10 million reads per sample. Through alignment to version v3.22 of the B73 genome, 25610 and 30057 expressed annotated transcripts were detected in MLs and ILs, respectively. From these, 1112 ML and 691 IL annotated genes showed differential expression between SD and NB treatments [false discovery rate (FDR)=0.05; Supplementary Table S1]. More than half of these transcripts, 756 MLs and 522 ILs, showed increased levels upon floral induction. GO term enrichment was conducted on these differentially expressed genes (Fig. 2). In MLs, this revealed up-regulation of genes involved in oxidation/reduction status, phospholipid metabolism and carbohydrate metabolism, and down-regulation of glycosyl hydrolase genes. In ILs, significant GO terms up-regulated included microtubule transport, xylosyl transferase activity, glycosyl hydrolase activity, and alterations to the cell wall and apoplast. In ILs, down-regulated GO terms included peptidase inhibition and organic acid metabolism.

Maize population genetics studies have revealed numerous small effect loci underpinning flowering time variation (Buckler *et al.*, 2009; Coles *et al.*, 2010; Li *et al.*, 2016; Romero Navarro *et al.*, 2017). A candidate gene list was created to compare putative floral regulators with observed differentially expressed genes (Supplementary Table S2) (Buckler *et al.*, 2009; Coles *et al.*, 2010; Dong *et al.*, 2012; Li *et al.*, 2016). Several genes implicated in the floral transition were detected in MLs and ILs, and a subset exhibited differential expression (Table 1; Fig. 2B). These included *ZCN* genes related to maize florigens. In MLs, up-regulation of *ZCN7*, *ZCN8*, *ZCN12*, and *ZCN15*, and down-regulation of *ZCN18* and *ZCN26* was observed in response to SDs. Genes associated with the circadian clock showed significant expression differences. In induced MLs, expression of *CIRCADIAN CLOCK ASSOCIATED1* (*CCA1*), and a *CCT-like* gene was increased, while an *EARLY FLOWERING3-like* (*ELF3-like*) homologue, and another *CCT-like* gene decreased in expression. A homologue of *PHYTOCHROME INTERACTING FACTOR 3* (*PIF3*) showed decreased expression in SD ILs. Notable clock components that showed no altered expression in induced versus uninduced teosinte include *ZmCCT* and *CONZ1*.

**Table 1.** Genes of interest differentially expressed in vegetative/reproductive (night-break versus short day) immature (IL) and mature (ML) teosinte leaves

Gene identifier	Gene annotation	Log_2_ fold change (vegetative/ reproductive)
**Teosinte ILs**		
GRMZM2G180406	BHLH transcription factor	–0.608118
GRMZM2G039996	Chlorophyll A-B binding protein	–0.594586
GRMZM2G082520	Expansin precursor	*0.730048*
GRMZM2G095968	Expansin-like	–1.57643
GRMZM2G052616	GATA27	*0.74874*
GRMZM2G147716	MADS67	–**3.32879**
GRMZM2G032339	MADS4 (ZMM4)	–5.81219
GRMZM2G115960	PiF3	0.75819
GRMZM2G125441	Protodermal factor 1 homologue	–**0.681943**
GRMZM2G700665	Rap2.7	1.58665
GRMZM2G052616	GATA-27	*0.74874*
GRMZM2G025783	Kelch repeat protein	**–1.64136**
GRMZM2G465091	TCP family transcription factor	–1.71166
GRMZM2G413006	Xyloglucan GH16	**–** *0.849902*
GRMZM2G392125	Xyloglucan GH16	**–** *2.291*
GRMZM2G026980	Xyloglucan GH16 (XET1)	**–1.54506**
GRMZM2G038898	Xyloglucan GT14	**–1.47633**
GRMZM2G116079	Zinc finger family protein	**–0.828233**
**Teosinte MLs**		
GRMZM2G026223	Agamous-Like6 (SOC1 homologue)	0.690749
GRMZM2G369472	AP2-EREBP172	–**1.69213**
GRMZM2G400714	C2H2 zinc finger protein	**–2.40909**
GRMZM2G134023	C3HC4 zinc finger protein	**–2.10963**
AC225718.2_FG006	Calcium-binding EF-hand	**–2.08808**
GRMZM2G340807	Calcium-binding EF-hand	**–1.85258**
GRMZM2G106945	Calmodulin-related protein	**–1.59218**
GRMZM2G014902	CCA1	–0.836844
GRMZM2G155370	CCT-Like	–*1.09463*
GRMZM2G075562	CCT-Like	1.19754
AC233870.1_FG003	ELF3-Like	*0.605407*
GRMZM2G032339	MADS4 (ZMM4)	–2.58678
GRMZM2G347280	TRPP1 (Trehalose6P phosphatase1)	–*0.840243*
GRMZM2G425774	Vernalization5 (VEL1-Like)	0.588868
GRMZM5G871347	WRKY93	–**2.19404**
GRMZM2G141756	ZCN7	0 expression NB
GRMZM2G179264	ZCN8	–5.2493
GRMZM2G400167	ZCN26	**1.4505**

Log_2_ fold change is reported, such that negative values represent an increase in reproductive plants. Transcripts in common with B73 vegetative/reproductive plants are shown, with bold and italics fold change values representing congruent and opposite expression changes, respectively.

Transcriptional regulators associated with photoperiodic flowering were altered in SD versus NB teosinte leaves. *SUPPRESSOR OF OVEREXPRESSION OF CONSTANS 1* (*SOC1*) homologue *AGAMOUS-LIKE6* (*ZAG6* or *MADS56*) and a *VERNALIZATION5* (*VRN5*) homologue were both down-regulated in MLs of induced teosinte. An established floral repressor, *RAP2.7*, showed decreased expression in ILs (Salvi *et al.*, 2007; Castelletti *et al.*, 2014). *MADS4* (*ZMM4*), an *AP1* homologue that promotes the floral transition in temperate maize SAMs, had higher expression in both SD leaf tissues (Danilevskaya *et al.*, 2008). An uncharacterized gene, *MADS67*, had higher transcript accumulation in florally induced ILs. Differentially expressed genes were compared with putative domestication and improvement targets to find possible overlap between selection targets and genes altered by floral induction (Supplementary Table S3) (Hufford *et al.*, 2012). Differentially expressed genes in teosinte ILs (*P*=0.60 domestication, *P*=0.81 improvement; χ^2^ goodness of fit) and MLs (*P*=0.86 domestication, *P*=0.13 improvement; χ^2^ goodness of fit) showed no significant enrichment in targets of historical selection. Altered expression of several genes was confirmed by independent qPCR analysis (Supplementary Fig. S1). Transcript profiling of teosinte grown under SD and NB conditions is congruent with photoperiod pathways in other plants, where perception of SDs changes the circadian clock output and increases florigen levels.

### B73 leaf mRNA expression changes (WT versus *id1*) reveal alterations in circadian clock genes and carbohydrate utilization

Total RNA sequencing from B73 tissues produced an average of 10 million reads per sample and detected a similar number of expressed transcripts as detected in teosinte, namely 28115 and 29163 expressed annotated transcripts from MLs and ILs, respectively. In MLs and ILs, 1519 and 179 annotated genes, respectively, showed differential expression between the WT and *id1* (FDR=0.05; Supplementary Table S4). Of these, 926 in MLs and 135 in ILs were up-regulated upon flowering. GO enrichment of ML genes down-regulated upon flowering included organic acid metabolism and nitrogen compound metabolism (Fig. 2). GO terms enriched within up-regulated ML genes include oxidation/reduction status, hexosyl-transferase activity, and trehalose metabolism. Of 20 trehalose metabolism genes expressed in leaves (Henry *et al.*, 2014), seven were differentially expressed in MLs. *TREHALOSE-6-PHOSPHATE SYNTHASE1* (*TRPS1*), which synthesizes trehalose 6-phosphate (T6P), and *TREHALOSE-6-PHOSPHATE PHOSPHATASE1* (*TRPP1*), which degrades T6P, were increased and decreased, respectively, in WT MLs. Related to this finding, an *SNF-RELATED KINASE1* (*SnRK1*) regulatory β subunit gene showed decreased expression in *id1* IL tissues. Alterations in *SnRK1* and *T6P* regulation are consistent with perturbed carbon partitioning in *id1* tissues (Coneva *et al.*, 2007, 2012; Wong and Colasanti, 2007). In IL samples, only two GO terms were enriched: glycosyl hydrolase activity and iron ion binding, which were up- and down-regulated, respectively. In agreement with a previous microarray study, a severe reduction in transcript abundance of *DHURRINASE*-*like β-GLYCOSIDASES* was observed in *id1* ILs. Three *GLYCOSYL-HYDROLASE FAMILY 16* (*GH16*) genes and a *GLYCOSYL-TRANSFERASE FAMILY 14* (*GT14*) gene involved in hemicellulose remodelling showed altered expression in ILs. Transcriptome profiling of B73 flowering versus non-flowering leaves suggests changes in T6P metabolism, *SNRK1* regulation, and hemicellulose metabolism. This is consistent with previously observed alterations in leaf carbon utilization and carbon signalling upon disruption of ID1-mediated autonomous induction (Coneva *et al.*, 2012).

The list of candidate flowering time genes was compared with the differentially expressed genes (Fig. 2b; Table 2). Unlike teosinte, where six *ZCN* genes responded to floral induction, only *ZCN26* was differentially expressed in the B73 ML transcriptome profile. In contrast to a florigenic signal, *ZCN26* expression was down-regulated upon flowering. Expression analysis at the *ZCN8* locus is complex due to the paralogue ZCN7 (94.3% amino acid identity) and previously detected sense and antisense unspliced transcripts (Meng *et al.*, 2011; Mascheretti *et al.*, 2013, 2015). Therefore, strand-specific reads were remapped and quantified for either *ZCN7* or *ZCN8* spliced transcripts and *ZCN7* or *ZCN8* unspliced transcripts (Supplementary Fig. S2). *ZCN8* expression was reduced in B73 *id1* MLs, as previously reported (Meng *et al.*, 2011; Mascheretti *et al.*, 2013, 2015). This confirmed that *ZCN7* and *ZCN8* expression from B73 *Id1*^*+*^ MLs was lower than in teosinte SD MLs, consistent with observed differences in the teosinte transcriptome profile.

**Table 2.** Genes of interest differentially expressed in vegetative/reproductive (*id1* versus WT) immature (IL) and mature (ML) B73 leaves

Gene identifier	Gene annotation	Log_2_ fold change (vegetative/ reproductive)	ID1-binding site ≤2 kb upstream?
**B73 ILs**			
GRMZM2G069146	AP2-EREBP115	–2.04479	No
AC206951.3_FG017	AP2-EREBP182	–1.29962	**Yes**
GRMZM2G177220	ARR B6	–0.6696	No
GRMZM2G367834	CCT-Like	–0.479088	**Yes**
GRMZM2G162505	Chitinase2	2.04718	**Yes**
GRMZM2G076946	Dhurrinase-like β-glucosidase	–9.89604	**Yes**
GRMZM2G077015	Dhurrinase-like β-glucosidase	0 expression *id1*^*−*^	No
GRMZM2G014844	Dhurrinase-like β-glucosidase	–0.744816	No
GRMZM2G082520	Expansin precursor	***–*** *0.968866*	No
GRMZM2G052616	GATA27	–*0.736017*	**Yes**
GRMZM2G011357	ID1	–3.06022	No
GRMZM2G320287	IDD7	1.26634	**Yes**
GRMZM2G147716	MADS67	**–5.10897**	**Yes**
GRMZM2G125441	Protodermal factor 1 homologue	–**0.600682**	**Yes**
GRMZM2G144782	RING and CHY zinc finger protein	**–1.70151**	No
GRMZM2G011078	SCAMP	**–2.75259**	No
GRMZM2G025459	SNRK1 homolog	–0.912072	No
GRMZM2G413006	Xyloglucan GH16	*0.839937*	**Yes**
GRMZM2G392125	Xyloglucan GH16	*0.903474*	No
GRMZM2G026980	Xyloglucan GH16 (XET1)	**–1.22364**	**Yes**
GRMZM2G038898	Xyloglucan GT14	**–3.24967**	No
GRMZM2G116079	Zinc finger family protein	**–0.486814**	No
**B73 MLs**			
GRMZM2G369472	AP2-EREBP172	–**1.15875**	No
AC233935.1_FG005	C2C2 zinc finger protein	0.5426	**Yes**
GRMZM2G400714	C2H2 zinc finger protein	**–0.900848**	No
GRMZM2G134023	C3HC4 zinc finger protein	**–0.635558**	No
AC225718.2_FG006	Calcium-binding EF-hand	**–0.631169**	No
GRMZM2G340807	Calcium-binding EF-hand	**–0.882565**	No
GRMZM2G106945	Calmodulin-related protein	**–1.27029**	No
GRMZM2G155370	CCT-like	*0.519004*	**Yes**
GRMZM2G095598	CCT-like	–0.481031	No
GRMZM2G038783	CCT-like	–0.580766	No
GRMZM2G148772	CCT-like	–0.706497	No
GRMZM2G092363	CCT-like	–0.926503	**Yes**
GRMZM2G013398	CCT-like	0.756804	No
GRMZM2G179024	CCT-like	–0.466114	No
GRMZM2G405368	Conz1	–0.872319	**Yes**
GRMZM2G077015	Dhurrinase-like β-glucosidase	–5.46369	No
AC233870.1_FG003	ELF3-like	–*0.443462*	**Yes**
GRMZM2G171365	MADS1 (SOC1 homologue)	1.03822	**Yes**
GRMZM2G347280	TRPP1 (Trehalose6P phosphatase1)	*0.488956*	No
GRMZM2G068943	TRPS1 (Trehalose6P synthase1)	–1.2737	**Yes**
GRMZM5G871347	WRKY93	**–0.93739**	No
GRMZM2G400167	ZCN26	**0.9708**	No

Log_2_ fold change is reported, such that negative values represent an increase in reproductive plants. Transcripts in common with teosinte vegetative/reproductive plants are shown, with bold and italics fold change values representing congruent and opposite expression changes, respectively.

Significantly altered transcription factor genes included the floral regulator *MADS1* (a *SOC1* orthologue) that was down-regulated in MLs upon flowering. Additionally, *MADS67* was expressed more highly in WT ILs. Unexpectedly, although plants were grown under identical photoperiods, significant expression changes of circadian clock-associated genes were observed (Table 2). These clock genes were different from those affected by floral induction in teosinte. Nine *CCT-like* genes showed altered expression in florally induced plants. Six of these, including the putative maize *CONSTANS* orthologue, *CONZ1*, had increased transcript levels in WT MLs, and two *CCT-like* genes and a homologue of the Arabidopsis clock gene *LUX* had decreased levels. Expression of one *CCT-like* gene increased in WT ILs. Other genes linked to the circadian clock with altered expression include *ELF3-like*, *ELF4*, and a *PIF3* homologue. Like teosinte, *ZmCCT* genes showed no expression change. Expression of several genes was confirmed by qPCR (Supplementary Fig. S3). Genes differentially expressed upon floral induction were compared with putative domestication and improvement targets (Supplementary Table S3). Differentially expressed IL genes (*P*=0.58 domestication, *P*=0.88 improvement; χ^2^ goodness of fit) and ML genes (*P*=0.43 domestication, *P*=0.08 improvement; χ^2^ goodness of fit) showed no enrichment in putative selection targets. mRNA transcriptome changes associated with disruption of autonomous floral induction point to changes in circadian clock genes distinct from those observed in photoperiod induction.

To predict possible direct ID1 direct targets, sequences homologous to the consensus binding motif, TTTGTCSYWWT (IUPAC nucleotide code) were identified near differentially expressed genes (Supplementary Table S5; Kozaki *et al.*, 2004). ID1 may directly bind to and change the epigenetic state of genes in the ILs to modulate later gene expression in MLs (Mascheretti *et al.*, 2015). Therefore, a motif search was conducted ≤2 kb upstream of genes differentially expressed in both tissues (Supplementary Table S5). Several genes of interest contained putative binding sites near their promoters (Table 2). For IL genes, these included carbon metabolism genes such as a *DHURRINASE*-*like* gene, two *GH16* genes, a *CCT-like* gene, and *MADS67*. For putative targets in ML genes, sites were found upstream of *CONZ1*, two other *CCT-like* genes, an *ELF3-like* gene, *MADS1*, and *TRPS1*. Thus carbohydrate utilization and clock output genes may be components of the autonomous pathway directly controlled by ID1.

### Photoperiod-dependent teosinte and autonomously flowering maize share leaf transcriptional changes in response to flowering

**Fig. 3.**
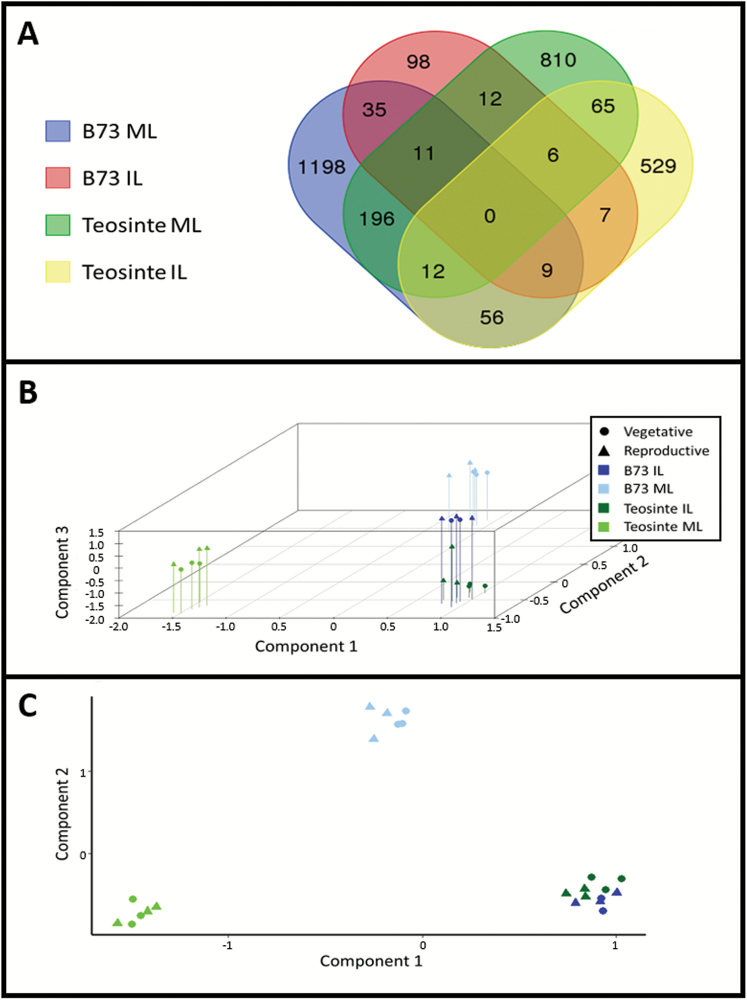
Comparison of immature leaf (IL) and mature leaf (ML) transcriptomes from induced and uninduced teosinte and B73. (A) Venn diagram displaying the number of transcripts differentially expressed in both reproductive/vegetative B73 (WT versus *id1*) and teosinte (short-days versus night-break). Principal component analysis (PCA) of B73 (blue) and teosinte (green) mRNA mature leaf (ML; light shades) and immature leaf (IL; dark shades) transcriptomes in three (B) and two (C) dimensions, capturing 86.3% and 78.4% of the variance, respectively. Reproductive treatments are depicted as triangles, and vegetative treatments as circles.

To examine universal changes accompanying the floral transition, transcripts altered in each group of florally induced versus uninduced tissue were compared (Fig. 3a). No genes were found to be differentially expressed in all treatment combinations. In MLs, 219 annotated transcripts were differentially expressed in teosinte and B73, which is more than expected due to chance (*P*<2.2E-16; χ^2^ goodness of fit). Of these, 141 transcripts show the same direction of change in reproductive and vegetative tissues. Some transcripts in common in MLs include *ZCN26*, *AP2-EREBP172*, *WRKY93*, Ca^2+^-sensitive genes, and two uncharacterized zinc finger genes. Of possible floral regulation genes, only *ZCN26* was commonly expressed in induced MLs. In ILs, 22 transcripts were differentially expressed in both teosinte and B73, which is more than expected due to chance (*P*<2.2E-16; χ^2^ goodness of fit). Eleven transcripts showed common expression changes upon induction. These included *MADS67* and a homologue of Arabidopsis *PROTODERMAL FACTOR1* (*PDF1*). The four aforementioned *GH16*/*GT14* hemicellulose remodelling genes were differentially expressed in both teosinte and maize ILs, comprising 18% of the overlap in this tissue. These genes are a part of the GO term ‘hydrolase activity, acting on glycosyl bonds’, which is up-regulated in both teosinte and B73 IL data sets. In MLs, the predominant common GO term is oxidative/reductive status, suggesting that large-scale leaf redox changes accompany the floral transition in domesticated maize and wild teosinte. These commonly regulated genes, however, include few of the canonical floral regulators differentially expressed in teosinte or B73 leaves. Even so, the comparison of gene expression in florally induced versus uninduced teosinte and B73 leaves unveiled a significant overlap in the transcriptional response to the floral transition.

PCA of all samples showed that, when three principal components are used, the groups separate into four clusters that correspond to each genotype–tissue combination (Fig. 3b). For two components, however, the teosinte IL and B73 IL transcriptomes cluster together (Fig. 3c), illustrating that differences between teosinte and B73 transcriptomes is greater in MLs than in ILs. No clustering is observed between reproductive or vegetative groups, showing that fewer transcriptional changes accompany the floral transition than those related to genotype and tissue. This is consistent with the relatively few transcripts (10^2^–10^3^) altered by induction in both genotypes.

### Floral induction affects miR399 expression in both teosinte and B73 leaves

**Fig. 4.**
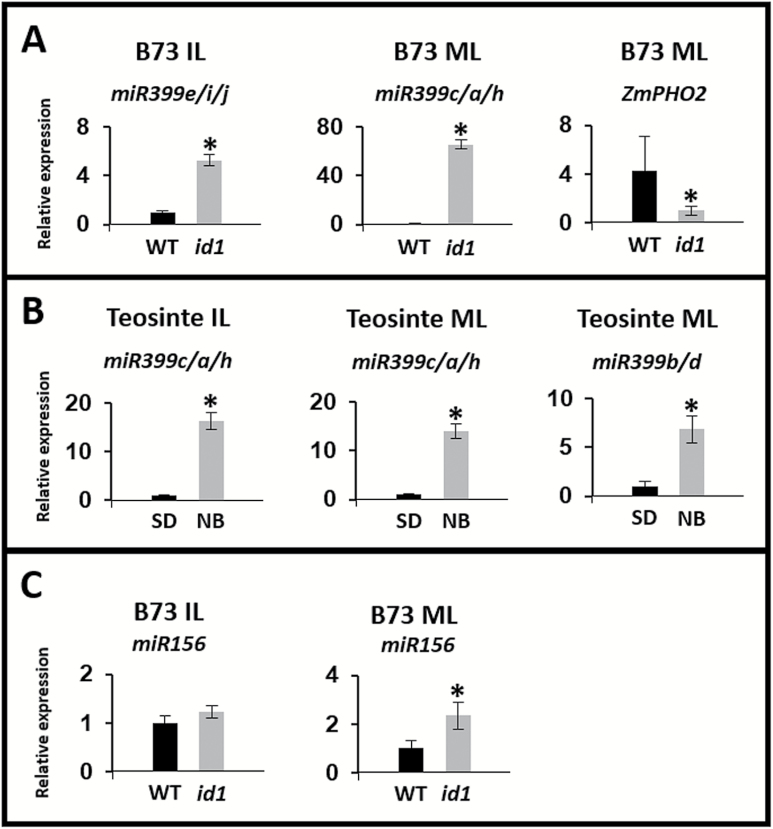
qPCR confirmation of miRNA and miRNA target gene expression changes upon floral induction. (A) Stem–loop qPCR quantification of *miR399* isoforms and its target *ZmPHO2* from immature (IL) and mature (ML) leaves of maize B73 WT and *id1* plants. (B) Stem–loop qPCR quantification of *miR399* isoforms in teosinte plants grown under inductive short days (SD) and inhibitory night breaks (NB). (C) Stem–loop qPCR quantification of *miR156* expression in ILs and MLs from B73 WT and *id1* plants. The primers used are not specific for a unique miRNA isoform, and a group of isoforms detected is reported. These graphs are representative of data obtained for one of the three biological replicates analysed. An asterisk indicates a statistically significant change (*P*≤0.05) when it was achieved across biological replicates.

sRNA sequencing performed in tandem with mRNA sequencing revealed changes in sRNA species that may regulate genes controlling the floral transition in SD versus NB teosinte and WT versus *id1* leaves. Ten million 50 bp single-end reads per sample were generated for each genotype and tissue. This analysis detected 119269 clusters producing unique sRNA from the B73 data sets and 469533 clusters of sRNA in teosinte (Supplementary Tables S7–S9). Annotated maize miRNA sequences were used to quantify miRNA expression levels (Supplementary Tables S7, S10). The only miRNAs with significant levels in *id1* leaves were *miR399c* and *miR399e* in MLs and ILs, respectively. These *miR399* isoforms were more abundant in non-flowering tissue than in the WT (Supplementary Table S7). In support of its established role in modulating gene expression, the previously confirmed *in vivo* target of *miR399*, *GRMZM2G381709* (Lunardon *et al.*, 2016), an Arabidopsis *PHOSPHATE2* (*PHO2*) orthologue (hereafter *ZmPHO2*), had decreased mRNA abundance in *id1* B73 IL and ML tissues (Supplementary Table S4). This was confirmed by qPCR (Fig. 4). Teosinte also had differentially expressed *miR399* genes with increased accumulation in uninduced NB tissue. Differentially expressed *miR399* isoforms included *miR399c*, *miR399b*, and *miR399f* in MLs, and *miR399h* in ILs. Stem–loop qPCR confirmed the differential expression of these *miR399* isoforms in flowering and non-flowering tissues (Fig. 4). *miR156*, a known repressor of the reproductive transition, was among the differentially expressed annotated miRNAs in teosinte MLs and ILs (Supplementary Table S10; Wu and Poethig, 2006; Wu *et al.*, 2009). Isoforms *miR156e*, *miR156i*, and *miR156l* had increased expression in teosinte NB ILs. Stem–loop qPCR also demonstrated a small, but significant increase of *miR156* in *id1* issues (Fig. 4c).

## Discussion

### Teosinte and B73 leaf mRNA transcriptomes reveal responses to the floral transition by parallel pathways

During the domestication of temperate maize, autonomous floral inductive signals superseded the obligate photoperiod signals required by its tropical ancestor, teosinte. Here, leaf expression profiles of maize and teosinte at the floral transition were analysed to investigate central components of floral inductive pathways in *Z. mays*. These analyses revealed little overlap in the expression changes of many distinct floral regulators in teosinte and B73. This supports the hypothesis that teosinte possesses elements of an autonomous inductive pathway that act separately in the leaf, but are repressed under LDs. In this model, two events occurred during domestication: first, loss of dominant floral repressors, including *ZmCCT*, that allowed flowering without SD induction; and, secondly, potentiation of an extant autonomous inductive pathway (Yang *et al.*, 2013). This is consistent with other models proposed in recent studies (Dong *et al.*, 2012; Romero Navarro *et al.*, 2017). In both teosinte and B73 temperate maize, for both tissues sampled, only one candidate floral regulator, *ZCN26*, shows the same expression profile. Other transcripts showing conserved expression changes are involved in the response to floral induction, and may contribute to physiological responses that prepare the plant for reproductive growth.

### Expression of shared and distinct ZCN and MADS transcription factor genes is linked to photoperiodic and autonomous floral induction

In teosinte, the *ZCN8* gene shows dramatic induction in leaves of SD-grown plants, analogous to expression changes in florigen genes of photoperiod-responsive rice and Arabidopsis (Lazakis *et al.*, 2011; Meng *et al.*, 2011). Similarly, leaf expression of *ZCN7*, *ZCN12*, and *ZCN15* increases under inductive conditions in teosinte, demonstrating that these *FT* homologues also possess photoperiod sensitivity. The *ZCN7* sequence and expression pattern are nearly identical to those of *ZCN8*, and *ZCN15* is associated with floral timing in landrace populations (Romero Navarro *et al.*, 2017). However, whether any of these uncharacterized *ZCN* genes are *bona fide* florigens requires further study.

In contrast, *ZCN8* expression in leaves of B73 temperate maize grown under LD conditions showed more attenuated expression at the floral transition. Previous studies showed that *ZCN8* expression levels are 3- to 4-fold higher in SD- than in LD-grown B73; however, this is relatively modest compared with the enormous up-regulation of *ZCN8* in SD versus NB teosinte (Lazakis *et al.*, 2011). Moreover, *ZCN8* expression does not cycle in a diurnal pattern under LDs (Lazakis *et al.*, 2011; Meng *et al.*, 2011). *ZCN8* shows a slight increase in expression in WT versus *id1* MLs, downplaying its importance in the floral delay caused by *id1*. Transgenic overexpression or knock down of *ZCN8* causes minor variations in flowering time (1–4 leaves), whereas loss of *id1* causes an extreme flowering delay (Colasanti *et al.*, 1998; Meng *et al.*, 2011). If *id1* acts by activating *ZCN8* exclusively, loss of *ZCN8* expression should produce a comparable extreme flowering delay. These findings suggest that temperate maize retains a residual photoperiod response; however, *ZCN8* is not a major component of the autonomous pathway.

*ZCN26* had an altered response at the floral transition in leaves of both teosinte and maize. *ZCN26* expression is down-regulated in MLs in both cases, which is not indicative of florigen activity. Indeed, reduced *ZCN26* expression in MLs is consistent with anti-florigen activity (Pin *et al.*, 2010; Higuchi *et al.*, 2013). However, overexpression of *ZCN26* in transgenic maize was reported to have no effect on the floral transition (Meng *et al.*, 2011). Therefore, it will be interesting to determine the role played by *ZCN26* in leaves that accompanies flowering, but does not directly trigger the reproductive transition at the SAM.

*MADS* genes encode transcription factors that include members associated with flowering in diverse species (Mandel *et al.*, 1992; Danilevskaya *et al.*, 2008). *MADS67* is up-regulated in ILs of both teosinte and maize at the floral transition. *MADS67* has not been previously implicated in triggering reproductive growth, but there is evidence of selection during domestication and subsequent improvement of maize cultivars, indicating a possible role of agronomic importance (Hufford *et al.*, 2012). Paralogous genes *MADS1* and *ZAG6* were significantly down-regulated at the floral transition in MLs of B73 and teosinte, respectively. *MADS1* has an ID1 DNA-binding motif upstream of its transcription start site, perhaps indicating that MADS1 is a component of the autonomous pathway. A recent study identified *MADS1* as a *SOC1* orthologue that, when expressed in transgenic maize, altered flowering time in a bimodal fashion—low expression caused delayed flowering, but large increases resulted in earlier flowering by up to 2 weeks (Alter *et al.*, 2016). Consistent with these studies, *MADS1* showed a small increase in expression in *id1* MLs. *ZAG6* displayed expression changes in the same direction as *MADS1*, with a similar fold change in teosinte MLs under non-inductive NB conditions, suggesting that these paralogues have divergent functions, with both contributing to flowering but along different pathways. A maize whole-genome duplication event occurred between 5 and 12 million years ago (Blanc and Wolfe, 2004; Swigoňová *et al.*, 2004). Duplication of a *SOC1* precursor may have allowed for subfunctionalization of descendant genes into different floral inductive pathways, one of which was required for temperate growth habits.

### miR399 is down-regulated at the floral transition in teosinte and maize

Expression of *miR399* isoforms is higher in pre-transition leaves of both teosinte and B73. *miR399* targets *ZmPHO2*, which is implicated in regulating root phosphorus acquisition (Lunardon *et al.*, 2016). A genome-wide association study (GWAS) of flowering in maize landraces reported that *ZmPHO2* is associated with the timing of male and female flowering, strengthening the argument for a role for *miR399* and *ZmPHO2* in maize reproduction (Romero Navarro *et al.*, 2017). Phosphate deficiency is accompanied by increases in soluble sugars, and increased sugar concentrations subsequently induce phosphate starvation responses (Ciereszko *et al.*, 2005; Karthikeyan *et al.*, 2007; Dasgupta *et al.*, 2014). Curiously, plants starved of phosphate and photosynthate do not accumulate *miR399*; thus, sugar levels supersede the *miR399* expression response to phosphate (Liu *et al.*, 2010). Therefore, *miR399* levels may respond to altered abundance of leaf soluble sugars rather than phosphorus limitation. As previous studies have focused on root metabolism, the detection of *miR399* expression differences in leaves upon floral induction may point to an undiscovered role for *miR399* in leaves (Fujii *et al.*, 2005; Pant *et al.*, 2008).

### Disruption of ID1-mediated autonomous floral induction supports a link between leaf carbon metabolism and flowering in temperate maize

Previous microarray and metabolomic studies suggest that *id1* regulates a critical node that links leaf carbon status with long-distance signals that trigger the floral transition (Coneva *et al.*, 2007, 2012; Wong and Colasanti, 2007). The present analysis supports these findings and provides new candidates downstream of *id1.* These include carbon metabolism and potential floral regulator genes with ID1 DNA-binding motifs in their putative promoters. Population genetic studies have uncovered loci showing signs of selection during domestication and improvement through classical breeding, but whether any of these loci were instrumental in transforming the reproductive timing mechanism is unknown. The *GH16*, *MADS67*, and *TRPP1* genes are differentially expressed in both induced teosinte and maize, and show signs of historical selection (Hufford *et al.*, 2012). Analysis of these genes may reveal additional molecular changes that facilitated the spread of maize to temperate regions.

T6P is a critical sensor of carbon availability that tunes growth and carbon utilization to sucrose availability (Figueroa and Lunn, 2016). In WT MLs, expression of the T6P biosynthesis gene, *TRPS1*, increased and expression of a gene involved in T6P catabolism, *TRPP1*, decreased. *TRPS1* is a possible target of ID1 because it has a consensus ID1-binding motif in its putative promoter. GO term enrichment for up-regulated transcripts in WT MLs also supports changes in T6P metabolism. This agrees with a previous metabolomic study that suggested increased T6P levels in B73 leaves at the floral transition (Coneva *et al.*, 2012). T6P levels are correlated with sucrose (Yadav *et al.*, 2014), yet *id1* MLs have higher sucrose levels and signatures of lower T6P, suggesting that *id1* affects both carbon sensing and partitioning. Consistent with changes in carbon metabolism, a *SnRK1* β subunit is down-regulated in *id1* mutant ILs. *SnRK1* activity is correlated positively with low cellular energy status and negatively with T6P levels (Bouly *et al.*, 1999; Polge *et al.*, 2008; Zhang *et al.*, 2009). Developing ILs are major carbon sinks in both vegetative and reproductive shoots. Higher *SnRK1* activity in induced ILs may lower energy utilization in maturing leaves, reducing leaf sink activity at a time when reproductive sinks are growing in strength. Carbohydrate sensing has a demonstrable effect on the floral transition in other plants (Corbesier *et al.*, 1998; Moore *et al.*, 2003; Wahl *et al.*, 2013). Perception of sugar levels and subsequent *id1* signalling would ensure that flowering occurs when energy levels are sufficient to support reproduction. In Arabidopsis, carbon signalling through T6P acts independently of photoperiod and *miR156* to control flowering (Wahl *et al.*, 2013). Interesting parallels are seen with *id1* mutants, as loss of ID1 activity represses SD-induced florigen production and shows little change in *miR156* expression (Lazakis *et al.*, 2011; Meng *et al.*, 2011; Mascheretti *et al.*, 2015). This implies that autonomous carbohydrate signalling may be similarly retained across the monocot–dicot divide.

### Developing leaves of *Z. mays* show altered expression of hemicellulose metabolism genes at the floral transition

Of the 22 genes showing altered expression at the floral transition in both B73 and teosinte ILs, four are predicted to remodel hemicellulose. Given heightened levels of carbohydrates in *id1* mutant leaves, these remodelling genes may be involved in carbon metabolism. Hemicellulose is a major sink which, in some plants, is broken down for carbon remobilization (Hoch, 2007; Lee *et al.*, 2007). Expanding maize leaves rapidly turn over select polymers during cell wall loosening (Gibeaut and Carpita, 1991). Aberrant polymer remodelling could limit carbon recycling during cell wall development, ‘locking’ carbon in the wall and preventing remobilization. Alternatively, changes in the maize apoplast may perturb IL function. Homologous cell wall remodelling genes in Arabidopsis are involved in phloem development (Bourquin *et al.*, 2002). Maize ILs rely exclusively on apoplastic export of sugar from MLs via the phloem to support growth (Evert and Russin, 1993). Altering the cell walls of symplastically isolated phloem would have severe consequences for export of sugars and information molecules, including florigen (Colasanti and Sundaresan, 2000). A connection between walls and flowering has been described in maize and sorghum. *Maize brown midrib* (*bm*) mutants display both altered leaf lignin content and altered flowering time (Vermerris and Mcintyre, 1999; Vermerris *et al.*, 2002). The mechanism underlying *bm*-mediated effects on flowering is unknown, but could involve events in the ILs. Mechanistic studies of monocot cell wall remodelling may unveil important physiological changes that accompany the floral transition.

### Photoperiodic and autonomous floral induction alter expression of distinct genes linked to the circadian clock

Transcriptional changes in clock genes accompany the perception of inductive photoperiods and the transition to reproductive growth. Established photoperiodic models involve output from the circadian clock that triggers the expression of florigen (Tiwari *et al.*, 2010; Dong *et al.*, 2012; Hung *et al.*, 2012; Romero Navarro *et al.*, 2017). Photoperiod-sensitive maize, such as teosinte, are induced to flower by long nights. NB treatment disrupts the inductive dark period yet does not affect the day length. Expression of components of the maize circadian clock, including *CCA1*, *ELF3-like*, *PIF3*, and *CCT-like* genes, changes in response to SD induction. Several clock genes have been identified in previous studies of the genetic control of maize flowering time (Buckler *et al.*, 2009; Coles *et al.*, 2010; Hung *et al.*, 2012; Yang *et al.*, 2013; Li *et al.*, 2016). Other clock components show no expression changes upon photoperiodic floral induction, suggesting that they are entrained to other stimuli. *ZmCCT* represses maize flowering under LDs and has been implicated in floral adaptation to temperate climates (Hung *et al.*, 2012; Yang *et al.*, 2013). This study finds that *ZmCCT* levels are unchanged under NB conditions in leaves, suggesting that *ZmCCT* expression is affected by day length, rather than night length, and that other repressors act upon flowering under NB treatment. The observed expression changes in *ZCN* genes upon exposure to SD supports a role for circadian clock output in triggering florigen expression.

**Fig. 5.**
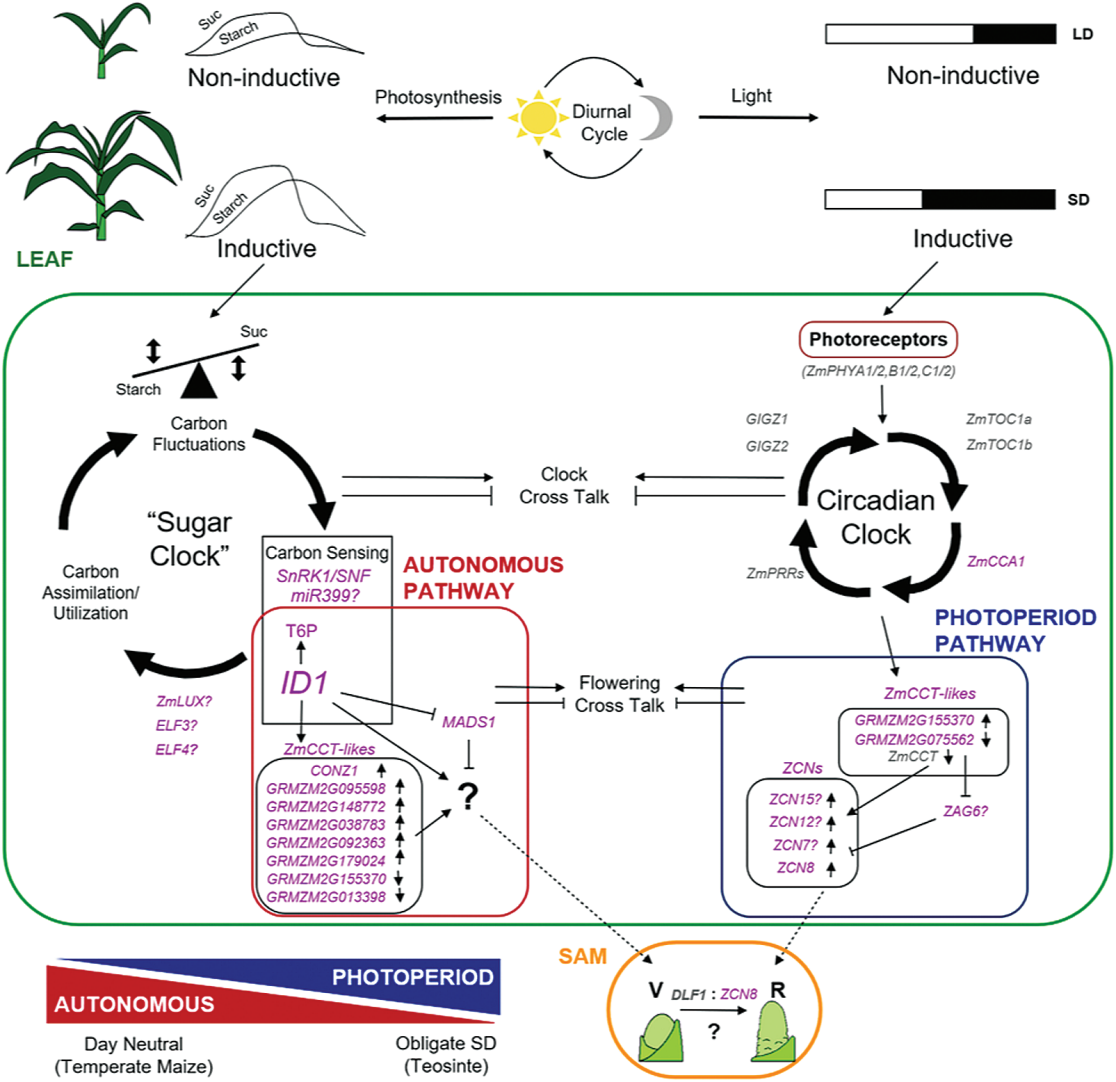
Model: parallel clocks in maize leaf regulate autonomous and photoperiod pathways that converge at the shoot apical meristem (SAM) to cause flowering. Diurnal cycles of light and dark (top) are perceived by photoreceptors to entrain the circadian clock and induce flowering under short-day (SD) photoperiods (blue box, right). Similarly, in growing plants, daily fluctuations of fixed carbon levels (starch and Suc) attain a critical threshold and feed into a proposed ‘sugar clock’ to activate autonomous induction (red box, left). Output from each clock affects expression of distinct *CCT-like* genes that produce florigens, such as the *ZCN* genes. For autonomous flowering, ID1 modulates input from carbon sensors to signal expression of *CCT-like* regulators and other possible florigens (indicated by ‘?’). Crosstalk between clocks integrates environmental and endogenous signals to balance plant growth and determine the relative contribution of each flowering pathway (illustrated by the red and blue triangle graphic, lower left). Genes found to have altered expression in this study are shown in purple; key genes implicated in flowering from other studies are shown in grey. Dotted lines indicate movement of florigens from leaves. The green boxed area represents activity in leaf.

Interestingly, many circadian clock output genes show altered expression in the WT versus *id1* comparison, including *CONZ1*, eight *CCT-like* genes, and an *ELF3-like* gene. Moreover, these clock-associated genes are different from those that are altered by photoperiod induction in teosinte. This was unexpected, as WT and *id1* plants were grown under identical photoperiods. Part of the circadian rhythm is entrained to daily changes in sugars due to photosynthesis rather than the perception of light though photoreceptors (Haydon *et al.*, 2013; Pattanayak *et al.*, 2015). In maize, sucrose and hexoses cycle diurnally (Kalt-Torres *et al.*, 1986). Consistent with altered carbon metabolism throughout the light/dark cycle, we propose that the cyclical sugar cycle, or ‘sugar clock’, is altered by loss of *id1* function (Fig. 5). Changes in sucrose levels over the day accompany the late flowering *id1* phenotype and, although a sugar cycle corresponding to the light/dark period persists, the magnitude of changes is significantly altered (Coneva *et al.*, 2012). In this model, the magnitude of fluctuating photosynthate levels increases as plants grow, regardless of day length. Carbon sensors, including T6P, signal when a critical threshold is attained, relaying this information to ID1. ID1 acts as a key integrator that modulates the output from the ‘sugar clock’, such as *CCT-like* gene expression, to promote florigen production (Fig. 5). The nature of these autonomous florigens, whether *ZCN*-related genes, metabolites, or a multifactorial signal, remains to be determined. Notably, SD versus NB treatments do not alter leaf sucrose and starch levels in teosinte leaves (Coneva *et al.*, 2012). Hence, gene expression changes observed in teosinte at the floral transition are in response to photoperiod and not altered sucrose levels. Distinct outputs from parallel internal oscillators may contribute to flowering by photoperiod or autonomous signalling.

### Conclusions

Transcriptome comparison of flowering pathways in obligate photoperiod-dependent teosinte and day-neutral autonomous temperate maize suggests that these two pathways operate through distinct leaf gene networks. Therefore, potentiation of the autonomous pathway combined with loss of LD floral repression accommodated the northward migration of maize. Intriguing shared responses were observed at the floral transition in teosinte and maize. Conserved *miR399* down-regulation in leaves upon flowering alludes to novel roles for *miR399* and *ZmPHO2* beyond controlling phosphate uptake. Transcriptional regulators show conserved expression alterations in both temperate maize and teosinte. These include *MADS67*, a gene previously identified as a target of maize domestication and improvement. Furthermore, paralogues *MADS1* and *ZAG6* probably underwent subfunctionalization after prehistoric polyploidization, resulting in their involvement in the autonomous and photoperiodic pathways, respectively. Unique components of the circadian clock show mutually exclusive expression trends upon disruption of autonomous or photoperiodic pathways, suggesting that distinct components of the clock influence flowering via each pathway. This study illuminates genetic changes underpinning the evolution of temperate maize and provides new targets for single gene studies that will provide improved mechanistic understanding of the floral transition.

## Supplementary data

Supplementary data are available at *JXB* online.

Fig. S1. qPCR assays in teosinte.

Fig. S2. Expression from paralogues *ZCN7* and *ZCN8*.

Fig. S3. qPCR assays in maize B73.

Table S1. All annotated transcripts differentially expressed between teosinte treatment groups.

Table S2. Established candidate maize floral regulators.

Table S3. Genes common to historical selection events and differential expression upon floral induction.

Table S4. All annotated transcripts differentially expressed between B73 treatment groups.

Table S5. ID1-binding motifs ≤2 kb upstream of genes differentially expressed in *id1* tissues.

Table S6. Differentially expressed small RNA clusters between B73 treatment groups.

Table S7. Differentially expressed small RNA clusters between teosinte treatment groups.

Table S8. Differentially expressed microRNAs between teosinte treatment groups.

Table S9. List of primers used in qPCR.

Table S10. List of primers used in stem–loop qPCR.

Supplementary Table S1

Supplementary Table S2

Supplementary Table S3

Supplementary Table S4

Supplementary Table S5

Supplementary Table S6

Supplementary Table S7

Supplementary Table S8

Supplementary Table and Figure

Supplementary Legends
