# Supplementary material for: Distinct gene networks modulate floral induction of autonomous maize and photoperiod-dependent teosinte
Source: J Exp Bot. 2018 Apr 24;69(12):2937–52. doi: 10.1093/jxb/ery110 (PMC5972621; doi:10.1093/jxb/ery110)
Supplement: Supplementary Table and Figure [file ery110_suppl_supplementary_table_and_figure.pdf]

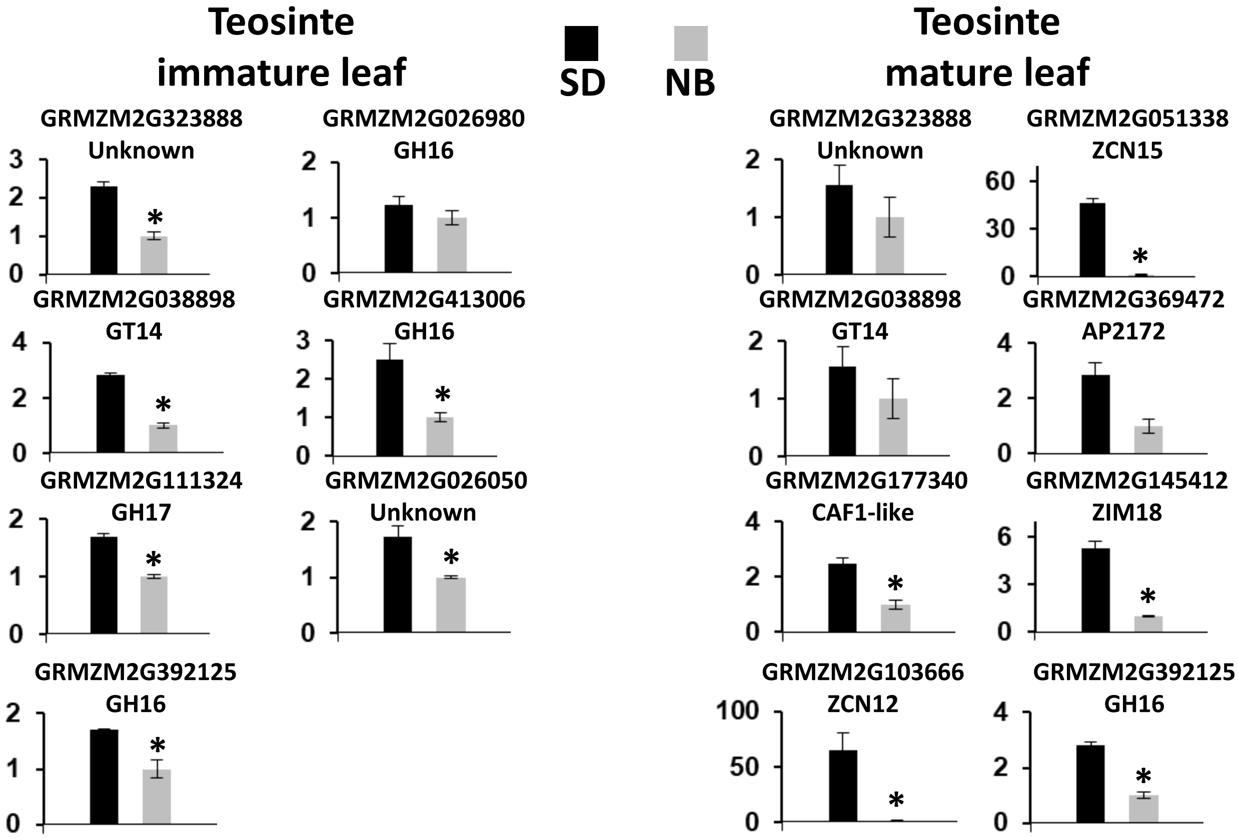

Figure S1 qPCR assays in teosinte. qPCR quantification of transcripts from immature and mature leaves of teosinte plants grown under inductive short days (SD) and inhibitory night breaks (NB) flowering conditions. These graphs are representative of data obtained for one of the three biological replicates analyzed. Asterisk indicates statistically significant change ($p \leq 0.05$) when it was achieved in the separate analysis of at least two of the three biological replicates.

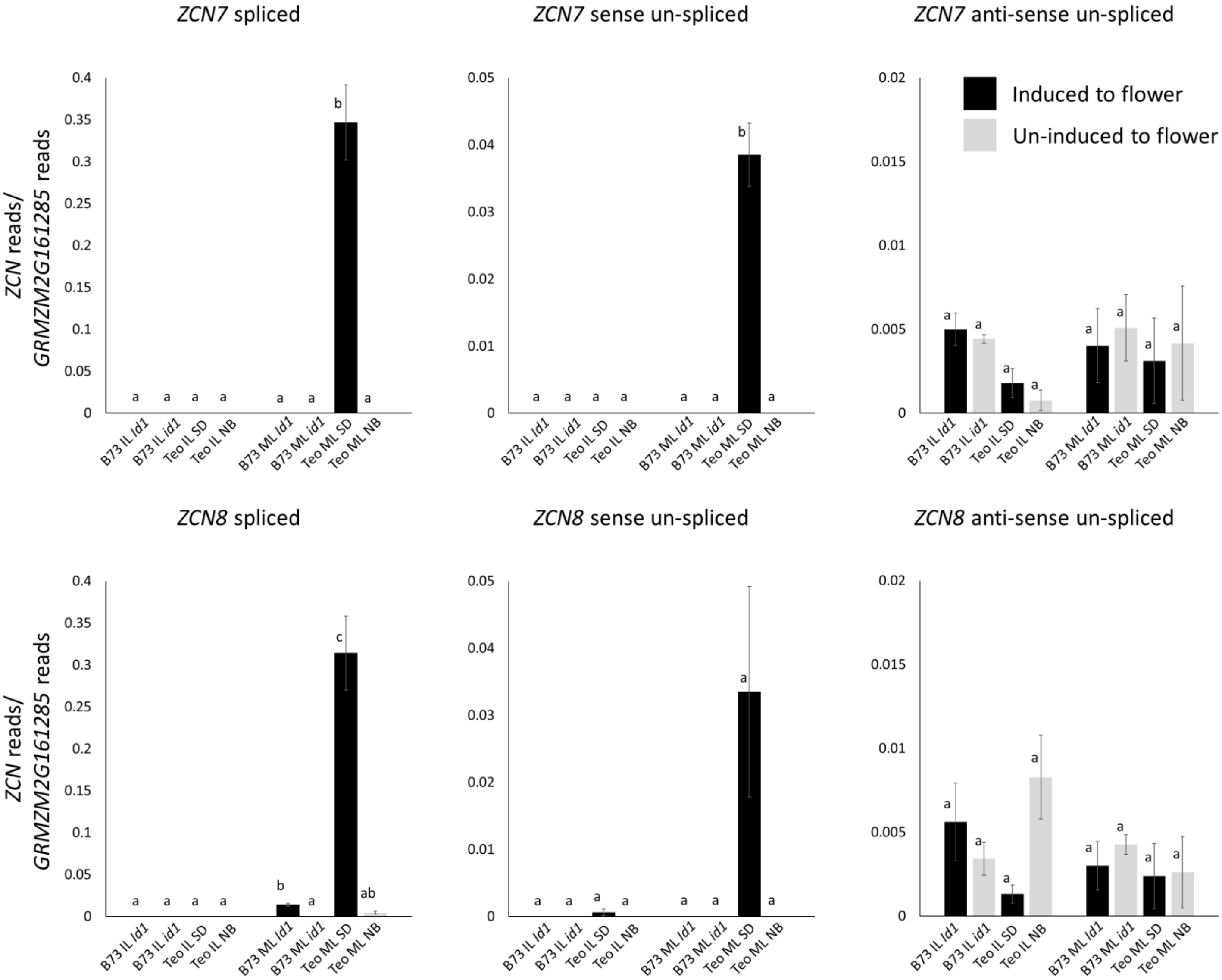

Figure S2 *ZCN7* and *ZCN8* mapped reads relative to reads mapped to *GRMZM2G161285*. Strand-specific reads from B73 and teosinte (Teo) immature and mature leaf (IL and ML) were mapped to *ZCN7* and *ZCN8* transcripts individually and standardized to the expression of *GRMZM2G161285*. Expression from induced B73 (*Id1*), induced short-day (SD) teosinte, uninduced B73 (*id1*) and uninduced night-break (NB) teosinte was determined for spliced sense, unspliced sense and unspliced anti-sense transcripts. MapQ quality scores were used to resolve reads mapped to both *ZCN7* and *ZCN8*. Reads mapping entirely to exons were removed from *ZCN7* and *ZCN8* unspliced sense transcript quantifications. Letters indicate statistically significant groups ($p \leq 0.05$), as determined through TukeyHSD (both anti-sense transcripts) and Welch t-tests (all other groups) after fdr adjustment for multiple comparisons. Error bars denote standard error.

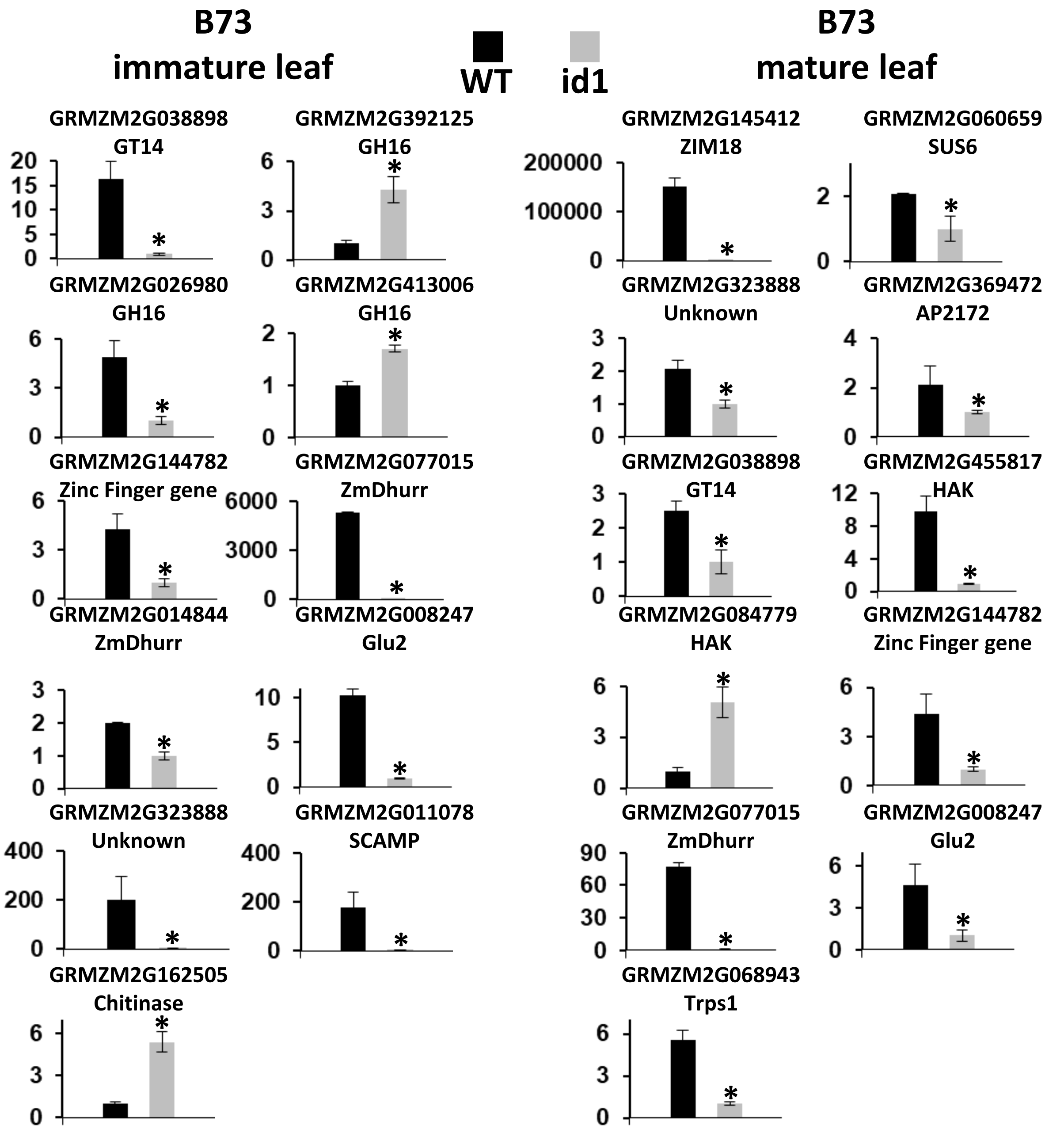

Figure S3 qRT-PCR assays in maize B73 inbred. qPCR quantification of transcripts from immature and mature leaves of maize B73 WT and *id1* mutant (*id1*) plants. These graphs are representative of data obtained for one of the three biological replicates analyzed. Asterisk indicates statistically significant change ($p \leq 0.05$) when it was achieved in the separate analysis of at least two of the three biological replicates.

Supplemental Table S9. List of primers used in qRT-PCR. a: Name of amplified sequence. b: sub-species. c: Forward (F) and reverse (R) primers reported in 5'-3' orientation.

**Sequence** [a]	**Sub-species**	**Primer combination** [b]
**GRMZM2G323888**	teosinte and maize	**F** CCTTCCACGACGCCATGT<br>**R** TACACAAAAGCGGGCGGA
**GRMZM2G144782**	teosinte and maize	**F** GTCACAAAATGCCAGGGAGATG<br>**R** TGAATAGCCCCACTAATCCTGC
**GRMZM2G038898**	teosinte and maize	**F** TCAAACAAACAGGCCGTTGAC<br>**R** AACAAAATTGGCTTTGGGCCG
**GRMZM2G026980**	teosinte and maize	**F** ACAACTACTGCACCGATAAGTACC<br>**R** TTCTACTCATCTACTACTTGGCCG
**GRMZM2G011078**	teosinte	**F** CAGTAGGGTTGGCGTCTTGT<br>**R** ACCTGGAGGGCTCAAAACAC
**GRMZM2G011078**	maize	**F** GCGACAGCGTCATTGTTGG<br>**R** CGCCTCATCTCCGCTTCTT
**GRMZM2G413006**	teosinte and maize	**F** TGATCTACAACTACTGCACCGAC<br>**R** ATCTCTGGTCTGAAGTGAACGAC
**GRMZM2G392125**	teosinte and maize	**F** TACTGCGCCGACACCAAG<br>**R** AATTGACCCGTGCGTCGT
**GRMZM2G154628**	teosinte	**F** CGCGGTCATCTACAACAAGG<br>**R** GGAAACAGAACAGGGCGTCA
**GRMZM2G111324**	teosinte	**F** CACGGATCTTGTGTTTATGCTGG<br>**R** ATTCTGCAAAGACTGCATGGTTC
**GRMZM2G026050**	teosinte	**F** CCTGGGTGGGTGAATAGGC<br>**R** AGCAGTTGACGATGGCAGAA
**GRMZM2G369472**	teosinte and maize	**F** CTGATGAGGCTGGACGAAGG<br>**R** TAGTTGAACGGCTGGGATCTG
**GRMZM2G177340**	teosinte	**F** GAGATGGCCAGGCTCTACTTC<br>**R** TGGAGCTCGAGGCCGTAG
**GRMZM2G145412**	teosinte and maize	**F** ATTCCTGTTCCGTGTCCCTAC<br>**R** ACGTGCAACTGTGTTTTTCAG
**GRMZM2G103666**	teosinte	**F** CATGGTGTTCGTGCTGTTCC<br>**R** TTGCAGTTGAAGTTGTGCCG
**GRMZM2G051338**	teosinte	**F** TCAACTGCCAGCGTGAGG<br>**R** CACGAGTACATCCTCCTGCC

**GRMZM2G122277**	maize	**F** ACATCAACATACCCCACATCCAC<br>**R** TCACCATATCATCATCAGGGCAG
**GRMZM5G840560**	maize	**F** CTCGTGGGTCATAATCTTTGTCATTT<br>**R** AATAGCTGACCTCATAGTTTGTGCTA
**GRMZM2G077015**	maize	**F** AAAGACGCAATAGACCTGGGAG<br>**R** ATGCCCGTCTTGTCTTTCTTCT
**GRMZM2G014844**	maize	**F** GTGTATGGCCGTGGGATGAA<br>**R** TGAACCCTTGGTGTTTGCCT
**GRMZM2G008247**	maize	**F** AGTGGTTAAAAGAGTTCAATACTGCG<br>**R** AGAATATGACTCCACTACCTCTCTCA
**GRMZM2G162505**	maize	**F** CGACCGCCCTGTGTTCTT<br>**R** TGGTCAGGGAGGAGACGG
**GRMZM2G060659**	maize	**F** GGACCAGATCAGTGTTTATTGTGA<br>**R** TCTCTTTACATCATCCAGCTCTCC
**GRMZM2G455817**	maize	**F** AGGCAGTCTACGCTGAGGA<br>**R** GTGAGGAGTGGGGTCTTGC
**GRMZM2G084779**	maize	**F** ACTGCTCCGGGTTGGAATG<br>**R** ACCACCTGCCTGCTTTGA
**GRMZM2G415359**	maize	**F** GCCTGTGATTTGCACGACATT<br>**R** TAGGCACCAGGAAAGCAACAA
**GRMZM2G141584**	maize	**F** ACAGCCCGGTTCAGTTCA<br>**R** CAGCCATCGTCCTCTTCTTC
**GRMZM2G381709**	maize	**F** ACGCATGATTGCTGGTGTT<br>**R** TCCCCTTGCCTTCGTATTT
**MEP**	teosinte and maize	**F** TGTACTCGGCAATGCTCTTG<br>**R** TTTGATGCTCCAGGCTTACC
**UBPC**	teosinte and maize	**F** CAGGTGGGGTATTCTTGGTG<br>**R** ATGTTCGGGTGGAAAACCTT

Supplemental Table S10. List of primers used in stem-loop qRT-PCR. See Materials and Methods and Varkonyi-Gasic et al. (2007) for details of the technology and meaning and use of primer type. Primers are written in 5'-3' orientation. UPL probe 21 reverse complement sequence is highlighted in bold.

**miR166**	**miRNA sequence**<br>UCGGACCAGGCUUCAUUCCC<br>**RT primer**<br>GTTGGCTCTGGTGCAGGGTCCGAGGTAT<br>TCGCAC**CAGAGCCA**ACGGGAAT<br>**forward primer**<br>GCAGCTTCGGACCAGGCTTC
**miR399e/i/j**	**miRNA sequence (miR399e)**<br>UGCCAAAGGAGAGUUGCCCUG<br>**RT primer**<br>GTTGGCTCTGGTGCAGGGTCCGAGGTAT<br>TCGCAC**CAGAGCCA**ACCAGGGCAA<br>**forward primer**<br>CGAACGGGTGCCAAAGGAGAG
**miR399c/a/h**	**miRNA sequence (miR399c)**<br>UGCCAAAGGAGAAUUGCCCUG<br>**RT primer**<br>GTTGGCTCTGGTGCAGGGTCCGAGGTAT<br>TCGCAC**CAGAGCCA**ACCAGGGCAA<br>**forward primer**<br>GCGACGGGTGCCAAAGGAGAA
**miR399b/d**	**miRNA sequence (miR399b)**<br>UGCCAAAGGAGAGCUGUCCUG<br>**RT primer**<br>GTTGGCTCTGGTGCAGGGTCCGAGGTAT<br>TCGCAC**CAGAGCCA**ACCTGGTC<br>**forward primer**<br>CCATGTCGTGCCAAAGGAGAGCT
**miR156**	**miRNA sequence**<br>UGACAGAAGAGAGUGAGCAC<br>**RT primer**<br>GTTGGCTCTGGTGCAGGGTCCGAGGTAT<br>TCGCAC**CAGAGCCA**ACGTGCTC<br>**forward primer**<br>GCGGCGGTGACAGAAGAGAGT
**miR166**	**miRNA sequence**<br>UCGGACCAGGCUUCAUUCCC<br>**RT primer**<br>GTTGGCTCTGGTGCAGGGTCCGAGGTAT<br>TCGCAC**CAGAGCCA**ACGGGAAT<br>**forward primer**<br>GCAGCTTCGGACCAGGCTTC
**Universal**	**Reverse primer** GTGCAGGGTCCGAGGT
