# Supplementary material for: Distinct gene networks modulate floral induction of autonomous maize and photoperiod-dependent teosinte
Source: J Exp Bot. 2018 Apr 24;69(12):2937–52. doi: 10.1093/jxb/ery110 (PMC5972621; doi:10.1093/jxb/ery110)
Supplement: Supplementary Legends [file ery110_suppl_supplementary_legends.docx]

**Supplementary Data**

**Figure S1**qPCR assays in teosinte

**Figure S2** Expression from paralogs *ZCN7* and *ZCN8*

**Figure S3** qPCR assays in maize B73

**Table S1**All annotated transcripts differentially expressed between teosinte treatment groups

**Table S2**Established candidate maize floral regulators

**Table S3**Genes common to historical selection events and differential expression upon floral induction

**Table S4**All annotated transcripts differentially expressed between B73 treatment groups

**Table S5**ID1 binding motifs ≤2kb upstream of genes differentially expressed in *id1* tissues

**Table S6**Differentially expressed small RNA clusters between B73 treatment groups

**Table S7**Differentially expressed small RNA clusters between teosinte treatment groups

**Table S8**Differentially expressed microRNAs between teosinte treatment groups

**Table S9**List of primers used in qPCR

**Table S10**List of primers used in stem-loop qPCR
